# Mitoquinone (MitoQ) Inhibits Platelet Activation Steps by Reducing ROS Levels

**DOI:** 10.3390/ijms21176192

**Published:** 2020-08-27

**Authors:** Diego Méndez, Diego Arauna, Francisco Fuentes, Ramiro Araya-Maturana, Iván Palomo, Marcelo Alarcón, David Sebastián, Antonio Zorzano, Eduardo Fuentes

**Affiliations:** 1 Thrombosis Research Center, Medical Technology School, Department of Clinical Biochemistry and Immunohaematology, Faculty of Health Sciences, Universidad de Talca, Talca 3460000, Chile; dmendez12@alumnos.utalca.cl (D.M.); darauna@utalca.cl (D.A.); malarcon@utalca.cl (M.A.); 2 Escuela de Medicina, Universidad de Talca, Talca 3460000, Chile; 3 Instituto de Química de Recursos Naturales, Universidad de Talca, Talca 3460000, Chile; raraya@utalca.cl; 4 Institute for Research in Biomedicine (IRB Barcelona), The Barcelona Institute of Science and Technology, 08007 Barcelona, Spain; david.sebastian@irbbarcelona.org (D.S.); antonio.zorzano@irbbarcelona.org (A.Z.); 5 Departament de Bioquímica i Biomedicina Molecular, Facultat de Biologia, Universitat de Barcelona, 08007 Barcelona, Spain; 6 Centro de Investigación Biomédica en Red de Diabetes y Enfermedades Metabólicas Asociadas (CIBERDEM), Instituto de Salud Carlos III, 28029 Madrid, Spain

**Keywords:** mitoquinone, mitochondria, platelets, ROS, MitoQ

## Abstract

Platelet activation plays a key role in cardiovascular diseases. The generation of mitochondrial reactive oxygen species (ROS) has been described as a critical step required for platelet activation. For this reason, it is necessary to find new molecules with antiplatelet activity and identify their mechanisms of action. Mitoquinone (MitoQ) is a mitochondria-targeted antioxidant that reduces mitochondrial overproduction of ROS. In this work, the antiplatelet effect of MitoQ through platelet adhesion and spreading, secretion, and aggregation was evaluated. Thus MitoQ, in a non-toxic effect, decreased platelet adhesion and spreading on collagen surface, and expression of P-selectin and CD63, and inhibited platelet aggregation induced by collagen, convulxin, thrombin receptor activator peptide-6 (TRAP-6), and phorbol 12-myristate 13-acetate (PMA). As an antiplatelet mechanism, we showed that MitoQ produced mitochondrial depolarization and decreased ATP secretion. Additionally, in platelets stimulated with antimycin A and collagen MitoQ significantly decreased ROS production. Our findings showed, for the first time, an antiplatelet effect of MitoQ that is probably associated with its mitochondrial antioxidant effect.

## 1. Introduction

The association between cardiovascular diseases (CVD) development and mitochondrial damage is well known [1]. This mitochondrial dysfunction leads to abnormalities in the respiratory chain, adenosine triphosphate (ATP) synthesis, and increased oxidative stress [2]. These abnormalities have also been evidenced in platelets from patients that present cardiovascular risk factors such as diabetes and cigarette consumption [3,4]. Several studies have shown that platelets that develop mitochondrial dysfunction display an enhanced production of reactive oxygen species (ROS) [5,6,7]. This mitochondrial ROS generation exerts a central role sensitizing the platelet to increase activation stimulated by a wide variety of agonists (e.g., thrombin, thrombin receptor activator peptide-6 (TRAP-6), U46619 and collagen) [8]. Following this the ROS produced in platelets activation stimulated by collagen leads to SHP-2 oxidation, which promotes tyrosine phosphorylation-mediated activation of phospholipase C and an increase in cytosolic calcium levels, among others [9]. Moreover, mitochondrial superoxide production can directly stimulate inflammasome-mediated platelet apoptosis [10].

Mitoquinone (MitoQ; 10-(4,5-dimethoxy-2-methyl-3,6-dioxo-1,4-cyclohexadien-1-yl)decyl triphenylphosphonium) is the pioneer molecule specifically designed to decrease mitochondrial oxidative stress that has been evaluated in clinical trials [11,12]. MitoQ is part of a redox system together with its reduced form, the hydroquinone MitoQuinol [13]. MitoQ is stored within mitochondria in vivo to prevent and protect the cellular damage induced by mitochondrial ROS overproduction and oxidative stress [14]. Fundamentally, this compound includes an ubiquinone moiety covalently adhered to a terminal aliphatic 10-carbon chain with a triphenylphosphonium complex (TPP^+^); that is the lipophilic cation that stores several hundred-fold in the mitochondria [15,16]. One of the most classical functions of this type of phosphonium salts is to increase the mitochondrial tropism of antioxidant molecules [17]; as over 95% of the cellular ROS produced by cells is the consequence of mitochondrial activity [18].

MitoQ is attached to the matrix-facing surface of the inner mitochondrial membrane (mainly in the hydrophobic core of the membrane) that is determined by the membrane potential, and is constantly reprocessed to ubiquinol by complex II of the respiratory chain [8]. The active part of MitoQ is ubiquinone (coenzyme Q10) [19]. The beneficial effects of MitoQ have been described in several disorders, such as diabetes, liver inflammation, and neurodegenerative diseases, among others [20]. Additionally, MitoQ is recommended as a therapeutic option for different CVD [21]. In this context, MitoQ has shown a protective effect against platelet mitochondrial dysfunction, decreasing the level of ROS in platelets and megakaryocytes, although its antiplatelet activity has not been fully described to date [22]. Therefore, in this study, the antiplatelet activity of MitoQ on human platelets was evaluated.

## 2. Results

**Figure 1:**
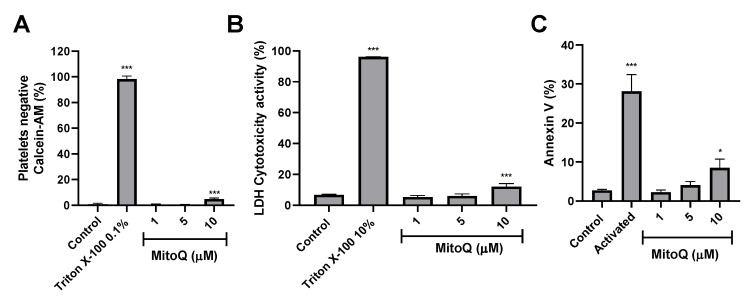
Cytotoxicity and phosphatidylserine exposure induced by MitoQ in platelets. (**A**) Platelet viability was evaluated by flow cytometry using calcein-AM. The populations of calcein-negative platelets (anti-CD61) were non-viable cells. (**B**) LDH release from platelets was analyzed with the LDH cytotoxicity assay kit in the supernatant and measured at 490 nm in a microplate reader. (**C**) Externalization of PS assessed by annexin-V binding in platelets stimulated by collagen plus TRAP-6 (activated) or MitoQ. (**A**) and (**C**) Platelets were identified as the CD61+ population and expressing the CD61 from these populations were analyzed in terms of percentage of the platelets with negative-calcein AM or annexin V. Representative dot plots of calcein-AM and annexin V assays were included as Supplementary Figures S1–S3. The statistical analysis was performed using the ANOVA (Tukey test). * *p* < 0.05, and *** *p* < 0.001.

The cytotoxicity of MitoQ on washed platelets by calcein-AM and LDH release are depicted in Figure 1. Washed platelets incubated with MitoQ 10 µM (4.8% ± 0.8%) significantly increased calcein-negative population (cytotoxic effect) compared to a non-treated control group (0.8% ± 0.8%; *p* < 0.001; Figure 1A). Likewise, it was observed that MitoQ 10 μM (12.1% ± 1.9%) induced significant cytotoxicity (an increase in LDH), referring to the basal control (6.7% ± 0.4%; *p* < 0.001; Figure 1B). To evaluate platelet apoptosis, the effect of MitoQ on PS exposure (PS binding to annexin V) was evaluated. As a positive control, washed platelets were incubated with the combination of two agonists (collagen plus TRAP-6). Thus the positive control (28.1% ± 4.3%) was significantly higher when compared to the basal control (2.7% ± 0.3%; *p* < 0.001). Similarly, it was observed that MitoQ 10 μM (8.5% ± 2.2%) induced a significant increase in PS exposure on the platelet membrane when compared to the basal control (*p* < 0.05), which suggests that MitoQ 10 μM could induce apoptosis on platelets. Meanwhile, in MitoQ at 1 and 5 μM there was no significant increase in PS on platelets (Figure 1C). From these results, it was decided to evaluate the antiplatelet effect of MitoQ up to a concentration of 5 μM, since it does not generate cytotoxicity activity or increase PS exposure on platelets.

**Figure 2:**
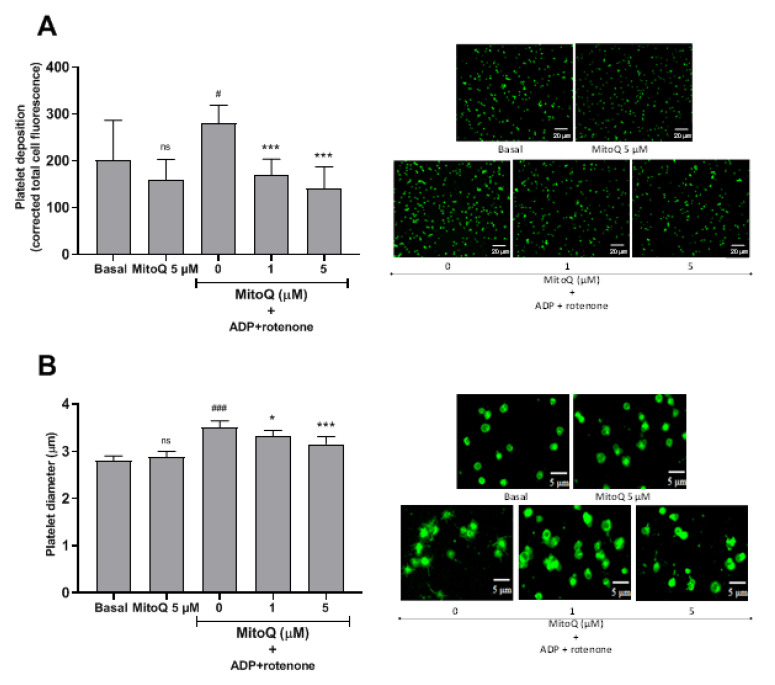
Effect of MitoQ on platelet deposition and spreading. Effect of MitoQ on platelet deposition (**A**) and spreading (**B**) on collagen-coated surfaces generated by mitochondrial dysfunction induced by rotenone plus ADP, and their representative images of each assay. The statistical analysis was performed using the ANOVA (Tukey test), ns: not significant, ^#^ *p* < 0.05 and ^###^ *p* < 0.001 vs. basal, and * *p* < 0.05 and *** *p* < 0.001 vs. activated platelets (ADP plus rotenone).

The effect of MitoQ on platelet activation steps, such as human platelet adhesion and spreading on collagen-coated surfaces, granule secretion, and aggregation was evaluated. As shown in Figure 2, platelets preincubated with rotenone 20 μM plus ADP 0.2 μM significantly increased platelet deposition and the diameter (spreading) on collagen-coated surfaces when compared with the control (basal; *p* < 0.05). Under these conditions, MitoQ 1 and 5 μM significantly decreased platelet deposition on collagen surfaces (*p* < 0.001; Figure 2A). Similarly, MitoQ 1 (3.3 ± 0.1 μm) and 5 μM (3.2 ± 0.2 μm), significantly decreased the diameter of the platelets when compared to the combination of rotenone 20 μM plus ADP 0.2 μM (3.5 ± 0.1 μm; Figure 2B). There was no statistically significant difference between MitoQ 5 μM and basal in both platelet deposition and spreading assays.

**Figure 3:**
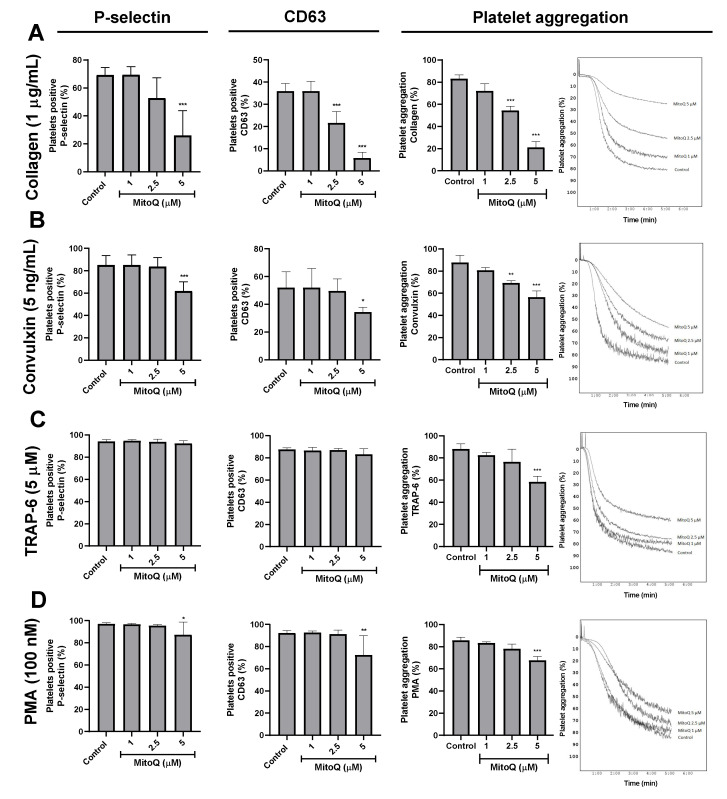
Effect of MitoQ on P-selectin and CD63 expression, and platelet aggregation. Effect of MitoQ on the expression of P-selectin and CD63, and platelet aggregation in human platelets stimulated by collagen (**A**), convulxin (**B**), TRAP-6 (**C**), and PMA (**D**). In P-selectin and CD63 assays, platelets were identified as CD61 + population and from these populations expressing the CD61 were analyzed in terms of percentage of the platelets with CD62 + (P-selectin) or CD63 +. Representative dot plots of P-selectin and CD63 assays were included as Supplementary Figures S6 and S7. The statistical analysis was performed using the ANOVA (Tukey test). * *p*< 0.05, ** *p* < 0.01 and *** *p* < 0.001.

Upon activation, exposure of P-selectin and CD63 was increased on the platelet membrane. As seen in Figure 3, the results showed that MitoQ decreased the expression of P-selectin and CD63 on platelet stimulated by collagen, convulxin, TRAP-6, and PMA. MitoQ 5 μM significantly decreased the expression of P-selectin from platelets stimulated by collagen (69.1% ± 5.5%), convulxin (85% ± 8.6%), and PMA (97.1% ± 0.9%) in the control group to 26% ± 17.7%, 62% ± 8.2%, and 87.1% ± 11.5%, (*p* < 0.05), respectively. MitoQ did not affect TRAP-6-induced P-selectin expression. Similarly, MitoQ 2.5 and 5 μM attenuated the effect of collagen-induced CD63 (35.9% ± 3.5%) expression to 21.6% ± 5.1% and 5.7% ± 2.6%, respectively (*p* < 0.05). Furthermore, MitoQ 5 μM significantly decreased the expression of CD63 stimulated by convulxin (52.1% ± 11.5%) and PMA (92.2% ± 2.4%) with values of 34.3% ± 3.4% and 72.4% ± 17.3%, respectively (*p* <0.05). Meanwhile, MitoQ did not affect TRAP-6 stimulated CD63 expression (Figure 3). The effects of MitoQ on platelet aggregation induced by different agonists (collagen, convulxin, TRAP-6, or PMA) are shown in Figure 3. It was observed that MitoQ exerted an antiaggregant effect on washed platelets stimulated by each agonist, but with differential inhibitory activities. Regarding platelet aggregation with collagen 1 μg/mL (83 ± 3.6%), MitoQ exerted a significant reduction of platelet aggregation at concentrations of 2.5 μM (54.3% ± 4.0%) and 5 μM (21.0% ± 5.3%; *p* < 0.001). Additionally, the platelet antiaggregant effect of MitoQ was confirmed on increasing collagen concentrations (Supplementary Figure S4). In convulxin (5 ng/mL)-induced platelet aggregation (87.7% ± 6.6%) MitoQ significantly decreased aggregation at concentrations of 2.5 (69.3% ± 2.1%) and 5 μM (56.3% ± 5.7%; *p* < 0.001), but with lower potency regarding collagen. In the activation with TRAP-6 5 μM (88% ± 4.74%) the antiaggregant effect was only observed by MitoQ at 5 μM (58.3% ± 5.1%; *p* < 0.001). Finally, the aggregation by PMA 100 nM (85.7% ± 2.9%) was only significantly decreased by MitoQ 5 μM (67.7% ± 3.5%; *p* < 0.001). Similarly, MitoQ inhibited collagen and ADP-induced platelet aggregation in PRP samples (Supplementary Figure S5).

**Figure 4:**
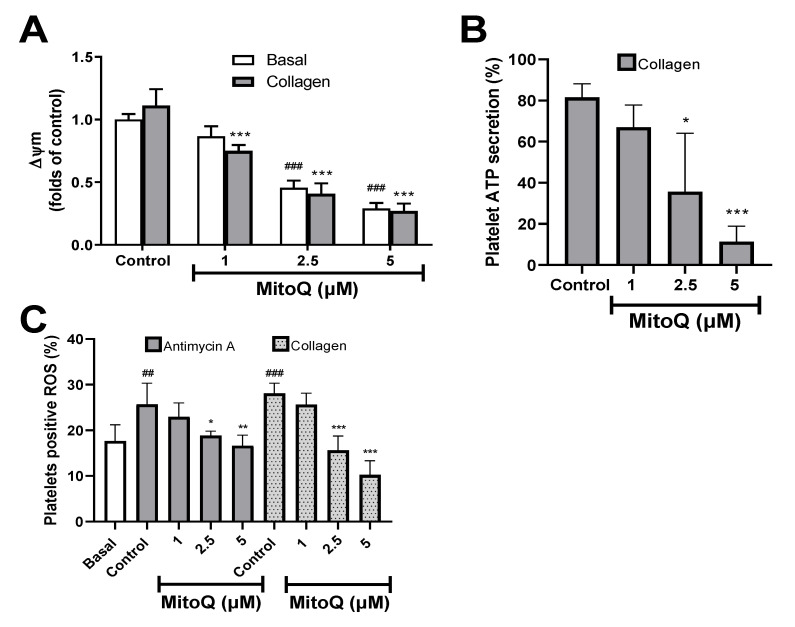
Effect of MitoQ on mitochondrial bioenergetics in collagen-stimulated platelets. (**A**) Mitochondrial membrane potential (∆Ψm) using TMRM was measured by flow cytometry. (**B**) Platelet ATP secretion was measured using chronolume reagent and expressed as a percentage. (**C**) Intraplatelet reactive oxygen species (ROS) generation was measured using DHE probe in a flow cytometer. (**A**) and (**C**) Platelets were identified as the CD61 + population and expressing the CD61 from these populations were analyzed in terms of change in mean fluorescence intensity from control (∆Ψm) and percentage of DHE-positive platelets (ROS production). Representative dot plots of ∆Ψm and ROS assays were included as Supplementary Figures S9 and S10. The statistical analysis was performed using the ANOVA (Tukey test). ^##^ *p* < 0.01 and ^###^ *p* < 0.001 vs. basal, and * *p* < 0.05, ** *p* < 0.01 and *** *p* < 0.001 vs. control.

Since MitoQ is recognized as a mitochondrial antioxidant, signaling studies were focused on platelet mitochondrial bioenergetics in the presence of collagen. As shown in Figure 4A, MitoQ 2.5 and 5 μM produced mitochondrial depolarization in unstimulated and collagen-stimulated platelets (*p* < 0.001). Likewise, collagen-induced platelet ATP secretion was inhibited by MitoQ 2.5 and 5 μM (*p* < 0.001; Figure 4B). Figure 4C shows the effect of MitoQ (1–5 μM) on the production of platelet ROS induced by antimycin A and collagen, quantified by the fluorescence of the DHE probe. A significant increase in ROS production was observed in platelets treated with antimycin A (25.7% ± 4.6%) and collagen (28.1% ± 2.2%) when compared with the basal state of platelets (17.7% ± 3.6%). Meanwhile, MitoQ at 2.5 and 5 μM produced a significant decrease in ROS production generated by antimycin A or collagen on platelets (*p* < 0.01). In MEF cells, treatment with antimycin A produced an increase in superoxide, measured by the Mitosox probe (fold change 1.45 ± 0.31), which was blocked by the addition of MitoQ 0.1 µM (fold change 0.79 ± 0.13; *p* < 0.05) (Supplementary Figure S8). Besides, the effect of MitoQ was also studied in a chronic oxidative stress model. Ablation of the mitochondrial fusion protein Mfn1 led to a chronic increase in ROS in MEF cells, which was not reduced by the MitoQ treatment (Supplementary Figure S8)

## 3. Discussion

In this article, the antiplatelet activity of MitoQ is reported for the first time. To evaluate compounds in platelets, it was necessary to study whether they had the cytotoxic capacity and to determine concentrations that adequately demonstrate their effect. The most common methods are centered on the quantification of the cytoplasmic activity of enzymes released by impaired cells and platelet viability by labeled with calcein-AM [23,24]. In this sense, we showed that MitoQ 10 μM was cytotoxic against platelets when evaluated by calcein-AM. Likewise in the LDH assay, MitoQ 10 μM caused damage to platelets, however platelets were unaffected at concentrations of 1 and 5 μM. This is in agreement with the results of LDH release, MitoQ 10 μM generated apoptosis in platelets (PS exposure), which reverted to the lower concentrations of MitoQ. With these data, it was decided to evaluate the antiplatelet effect of MitoQ in decreasing concentrations from 5 μM.

The activation of platelets comprises of different steps, such as adhesion, spreading, secretion, and aggregation [25]. Here it was shown that MitoQ inhibited the main steps of platelet activation. Thus platelet adhesion and spreading that are crucial steps for thrombosis were the processes by which platelets adhere and increase their contact area by deformation of the membrane at vascular injury sites [26]. ROS represents important secondary messengers in signal transduction cascades, which may be necessary for collagen-dependent platelet spreading [27]. Rotenone plus ADP to generate platelet spreading was used. Rotenone is an inhibitor of the transport of electrons in the mitochondrial complex I, it induces an increase in apoptosis and cell death; along with stimulating an overproduction of mitochondrial ROS [28]. Thus under these conditions, it activated platelets spread by filopodia and produced lamellipodia, causing a significant increase in platelet adhesion and surface area [29]. Microscopy experiments showed that MitoQ 1 and 5 μM significantly decreased platelet adhesion and spreading. After platelet activation, P-selectin and CD63 were translocated and expressed on the platelet surface membrane [30]. P-selectin expression was significantly decreased by MitoQ when platelets were activated with collagen, convulxin, or PMA. In the case of CD63, a similar effect was observed, where MitoQ significantly decreased collagen-, convulxin- and PMA-stimulated platelet activation. Platelet aggregation is one of the main steps in hemostasis and thrombosis [31]. Our results showed that MitoQ exerted an antiaggregant effect on platelets stimulated with four agonists, but with different potencies. Aggregation stimulated by collagen and convulxin was significantly decreased by MitoQ 2.5 and 5 μM. Different effects of MitoQ on collagen- and convulxin-mediated platelet aggregation could be due the fact that convulxin uses the p62/GPVI but not the alpha2beta1 integrin [32]. Additionally, the platelet antiaggregant effect of MitoQ on collagen concentrations demonstrated non-competitive behavior. In the case of TRAP-6 and PMA stimulated platelet aggregation, MitoQ only had a significant effect at 5 μM.

MitoQ induces important protection against oxidative stress-mediated mitochondrial dysfunction and apoptosis [33]. The elimination of ROS is achieved because MitoQ (in its reduced form) is oxidized and has a quick re-reduction at mitochondrial complex II [19]. Human and animal studies suggest that MitoQ (orally bioavailable that is rapidly absorbed into the bloodstream, it accumulates within mitochondria, and is non-toxic) protects against pathological alterations deriving from an increase in mitochondrial oxidative stress under different chronic disease conditions [11,34]. ROS production in platelets has been described as a critical step in the regulation of platelet activation [9]. In this context, in order to demonstrate the antioxidant effect of MitoQ on platelets, antimycin A as a ROS-inducing agent was used. Antimycin A impedes mitochondrial electron transport via its binding at the Qi site of complex III [35]. Our results showed that MitoQ 2.5 and 5 μM decreased the antimycin A-induced increase of intraplatelet ROS, which correlated with the antioxidant effect of MitoQ observed in MEF cells treated with antimycin A, as a model of acute oxidative stress. This was observed in a murine model of ROS-induced thrombocytopenia, where MitoQ normalized platelet production levels [22].

Since antimycin A inhibited collagen-induced platelet aggregation and secretion [36], the antiplatelet mechanism of MitoQ on mitochondrial bioenergetics in platelets stimulated by collagen was studied. The platelet activation induced by collagen was associated with mitochondrial ROS generation and this ROS boosted platelet activation by regulation of different pathways. Therefore platelets are both source and target of ROS [35,37,38,39]. Additionally, mitochondrial ROS can directly induce inflammasome-mediated caspase-dependent platelet death [10,40]. Here it was shown that human platelet exposure to MitoQ significantly decreased mitochondrial ROS production and PS was not perturbed. In platelets, the mitochondria were the main source of ATP generation through oxidative phosphorylation and that ATP alone or its breakdown to ADP may activate platelets [41]. In this context, as an additional antiplatelet mechanism, MitoQ decreased the intraplatelet ATP content [37,42,43]. However, mitochondrial depolarization induced by MitoQ could affect platelet contractility and diminish clot stability [8,44].

## 4. Materials and Methods

### 4.1. Preparation of Human Platelets

Platelets were obtained by phlebotomy from healthy donors (two weeks without drugs) that had previously accepted informed consent, as previously reported [23]. Briefly, whole blood was obtained with acid-citrate-dextrose (ACD) solution (proportion 4:1 *v*/*v*) and centrifuged at room temperature (RT) for 10 min × 200 *g* in order to collect platelet-rich plasma (PRP). Then PRP was centrifuged for 8 min × 900 *g*. Platelets pellet was suspended in calcium-free Tyrode’s buffer: ACD (proportion 5:1 *v*/*v*) and this was centrifuged again for 8 min × 900 *g* and platelets were resuspended in calcium-free Tyrode’s buffer and adjusted to the assays by a hematological counter (Mindray BC-3000 Plus, Japan) and used within 3 h for MitoQ (MedKoo Biosciences, Morrisville, NC, USA) assays.

### 4.2. Cytotoxic Activity

Washed platelets (200 × 10^6^ platelets/mL) were incubated with MitoQ (1, 5 and 10 µM) for 10 min at 37 °C. Ethanol 0.5% was used as a vehicle for MitoQ. After that, viability was determined by Accuri C6 flow cytometer (BD Biosciences, San Jose, CA, USA) using calcein-AM [24] and the supernatant was obtained by centrifugation at 900× *g* for 8 min and evaluated with lactate dehydrogenase (LDH) cytotoxicity kit (Cayman Chemical, Ann Arbor, MI, USA) [24]. A solution of Triton X-100 was used as a positive control for each cytotoxicity assay.

### 4.3. Phosphatidylserine (PS) Externalization

The externalization of PS in platelets was determined by the Accuri C6 flow cytometer [45]. Washed platelets (200 × 10^6^ platelets/mL) were preincubated for 5 at 37 °C min with CaCl_2_ (2 mM), and MitoQ (1, 5 and 10 µM) or positive control. The Annexin V positive control used was a combination of agonists TRAP-6 5 µM + Collagen 1 ug/mL. Then, 50 μL of the sample was diluted with 150 μL of annexin V binding buffer and incubated for 5 min. Finally, 30 µL of platelets were labeled with 2 µL of FITC Annexin V antibody and incubated in the dark for 30 min. The samples were acquired and analyzed in the Accuri C6 flow cytometer (BD Biosciences, San Jose, CA, USA).

### 4.4. Platelet Aggregation and ATP Secretion Assay

Platelet aggregation and ATP secretion were evaluated by light transmission and lumi-aggregometry, respectively (Lumi-aggregometer Chrono-Log, Haverton, PA, USA) [23]. Washed platelets (200 × 10^6^ platelets/mL) were previously incubated with CaCl_2_ (2 mM) and MitoQ (1, 2.5 and 5 µM) for 5 min at 37 °C. Following this, platelet aggregation was stimulated by collagen 1 µg/mL, convulxin 5 ng/mL, TRAP-6 5 µM or phorbol 12-myristate 13-acetate (PMA) 100 nM and was measured for 5 min at 37 °C. ATP secretion was detected using the chronolume reagent.

### 4.5. P-selectin and CD63 Platelet Expression

The effect of MitoQ (1, 2.5 and 5 µM) on P-selectin and CD63 platelet expression were measured by the Accuri C6 flow cytometer in washed platelets incubated with anti-CD62-PE or anti-CD63-PE for 30 min at RT. Platelets were identified with anti-CD61-FITC [23].

### 4.6. Platelet Adhesion and Spreading Assay

Washed platelets were preincubated with rotenone 20 μM plus adenosine 5′-diphosphate (ADP) 0.2 μM and were evaluated on collagen surface platelet deposition and platelet diameter using fluorescence microscopy (AXIO Examiner Z.1, Zeiss, Oberkochen, Germany). For this, a collagen film was prepared by heat fixation (37 °C) on a slide (collagen diluted in 0.02 M glacial acetic acid and suspended in distilled water). Then, 10 µL of platelet suspension previously incubated with the anti-CD61 FITC antibody was added to the collagen film. For platelet deposition, an objective 63×, in vivo camera system with the filter of the COLIBRI system at 470 nanometers (blue light), and 20% intensity was used. Nine images were randomly selected for each condition and fluorescence images were analyzed using ImageJ software (version 1.26t, NIH, USA). The platelet diameter was measured using the ZEISS ZEN imaging software. Using an increase of 200%, the horizontal diameter of 20 (random) platelets arranged in the field was measured in microns (µm).

### 4.7. Reactive Oxygen Species (ROS) Assay

ROS production was determined in washed platelets (50 × 10^6^ platelets/mL) using dihydroethidium (DHE) 10 µM [22]. The labeled platelets were pre-incubated with MitoQ (1, 2.5 and 5 µM) and then incubated with antimycin A 10 µM or collagen 10 µg/mL for 15 min at 37 °C to increase ROS levels. ROS formation was analyzed by the Accuri C6 flow cytometer [23].

### 4.8. Mitochondrial Membrane Potential

Mitochondrial membrane potential was determined using the potentiometric probe tetramethylrhodamine, methyl ester, perchlorate (TMRM) 100 nM, and analyzed by the Accuri C6 flow cytometer. MitoQ was evaluated at 1, 2.5 and 5 µM on washed platelets (50 × 10^6^ platelets/mL) and a control of mitochondrial depolarization carbonylcyanide-p-trifluoromethoxyphenylhydrazone (FCCP) 1 μM was used [23].

### 4.9. Determination of MitoQ Effects on Oxidative Stress in Mouse Embryonic Fibroblasts (MEF)

MEFwt and Mfn1KO cells were grown in DMEM 25 mM glucose, 10% FBS, and 1% penicillin-streptomycin. Cells were treated with MitoQ for 16 h. Superoxide anion was determined by incubating the cells with 50 nM MitoSox for 30 min. To analyze the effect of MitoQ 0.05 and 0.1 µM on acute oxidative stress, MEFwt cells were incubated with MitoSox in the absence or presence of 5 µM antimycin A. Cells were then washed in PBS1X twice, trypsinized and collected in 0.5 mL of medium. MitoSox fluorescence was quantified by flow cytometry (Gallios, Beckman Coulter).

### 4.10. Statistical Analysis

The obtained results were presented as the mean ± standard deviation (SD) and analyzed using Prism 8.0 software (GraphPad Inc., San Diego, CA, USA). Differences between three or more conditions were analyzed using ANOVA, and subsequently analyzed by Tukey’s post-hoc test. *p* values < 0.05 were considered significant.

## 5. Conclusions

In this study, an antiplatelet effect of MitoQ via inhibition of platelet adhesion and spreading, secretion, and aggregation has been described for the first time and this may be associated with its mitochondrial antioxidant effect. However, further research studies on animal models and in human clinical trials are needed to confirm the antiplatelet mechanism of MitoQ, which will evaluate potential applications (e.g., platelet storage) and therapeutic potentials.

## Supplementary Materials

The following are available online at https://www.mdpi.com/1422-0067/21/17/6192/s1, Figure S1: Representative dot plots of CD61+ (platelets), Figure S2: Representative dot plots of Calcein-AM positive platelets, Figure S3: Representative dot plots of annexin V positive platelets, Figure S4: Platelet antiaggregant effect of MitoQ on increasing collagen concentrations (0.1, 0.5, 1 and 2 µg/mL), Figure S5: Platelet antiaggregant effect of MitoQ in Platelet-Rich Plasma stimulated by collagen 1 µg/mL or ADP 4 µM, Figure S6: Representative dot plots of platelets P-selectin, Figure S7: Representative dot plots of platelets CD63+, Figure S8: MitoQ decreases intraplatelet ROS in MEFwt and Mfn1KO cells, Figure S9: Effect of MitoQ on mitochondrial membrane potential (∆Ψm), Figure S10: Effect of MitoQ on intraplatelet ROS generation.
