# Supplementary material for: Mitoquinone (MitoQ) Inhibits Platelet Activation Steps by Reducing ROS Levels
Source: Int J Mol Sci. 2020 Aug 27;21(17):6192. doi: 10.3390/ijms21176192 (PMC7503844; doi:10.3390/ijms21176192)
Supplement: Supplementary file 1 [file ijms-21-06192-s001.pdf]

SUPPLEMENTARY INFORMATION

Mitoquinone (MitoQ) Inhibits Platelet Activation Steps By Reducing ROS Levels

Diego Méndez [1,†], Diego Arauna [1,†], Francisco Fuentes [2,*], Ramiro Araya-Maturana [3], Iván Palomo [1,*], Marcelo Alarcón [1], David Sebastián [4,5,6], Antonio Zorzano [4,5,6] and Eduardo Fuentes [1,*]

[1] Thrombosis Research Center, Medical Technology School, Department of Clinical Biochemistry and Immunohaematology, Faculty of Health Sciences, Universidad de Talca, Talca 3460000, Chile; dmendez12@alumnos.utalca.cl (D.M.); darauna@utalca.cl (D.A.); ipalomo@utalca.cl (I.P.); malarcon@utalca.cl (M.A.); edfuentes@utalca.cl (E.F.)

[2] Escuela de Medicina, Universidad de Talca, Talca 3460000, Chile; panchoutd@gmail.com

[3] Instituto de Química de Recursos Naturales, , Universidad de Talca, Talca 3460000, Chile; raraya@utalca.cl

[4] Institute for Research in Biomedicine (IRB Barcelona), The Barcelona Institute of Science and Technology, Barcelona *08007*, Spain; david.sebastian@irbbarcelona.org (D.S.); antonio.zorzano@irbbarcelona.org (A.Z.)

[5] Departament de Bioquímica i Biomedicina Molecular, Facultat de Biologia, Universitat de Barcelona, Barcelona 08007, Spain

[6] Centro de Investigación Biomédica en Red de Diabetes y Enfermedades Metabólicas Asociadas (CIBERDEM), Instituto de Salud Carlos III, Madrid 28029, Spain

* Correspondence: edfuentes@utalca.cl (E.F.); panchoutd@gmail.com (F.F.); ipalomo@utalca.cl (I.P.)

† These authors contributed equally to this work

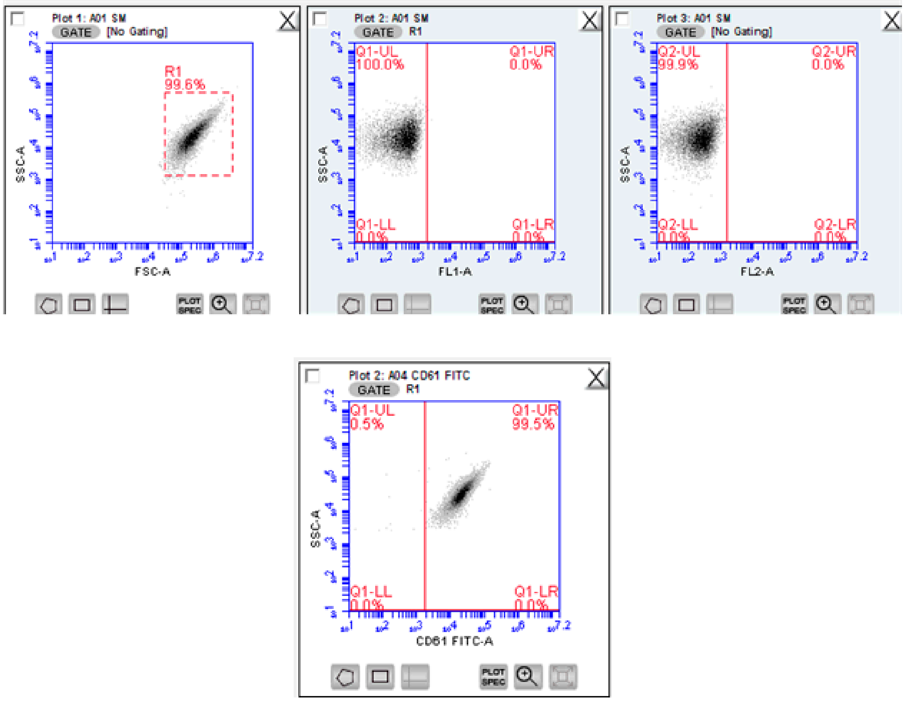

Figure S1. Representative dot plots of CD61+ (platelets).

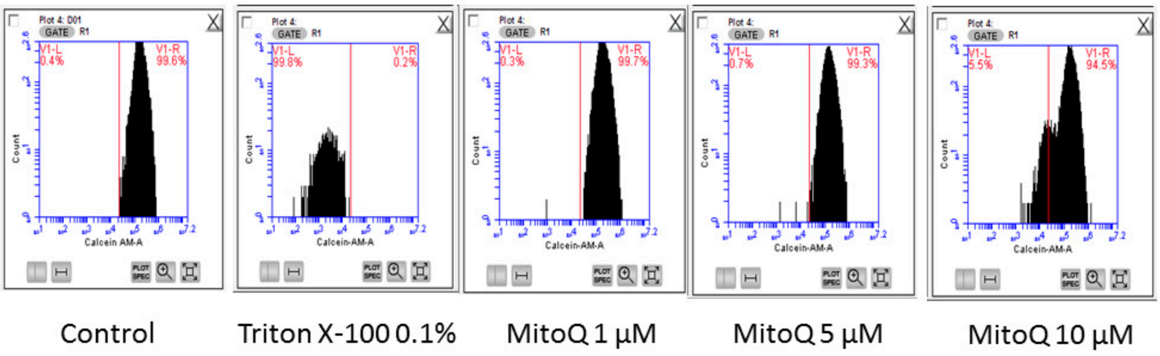

Figure S2. Representative dot plots of Calcein-AM positive platelets.

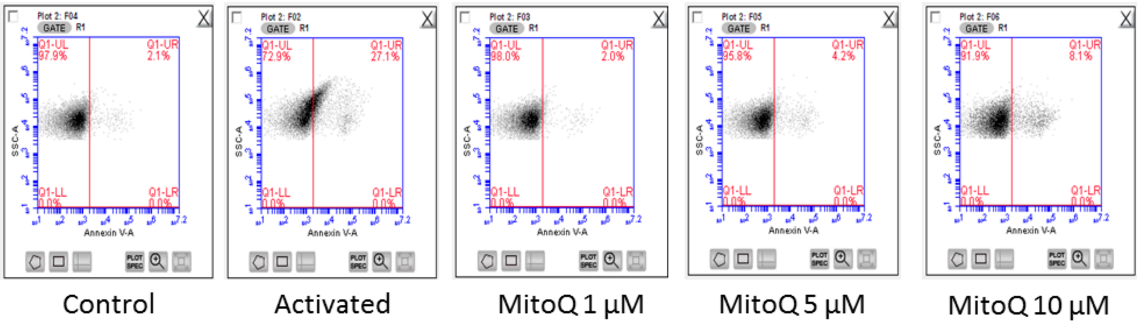

Figure S3. Representative dot plots of annexin V positive platelets.

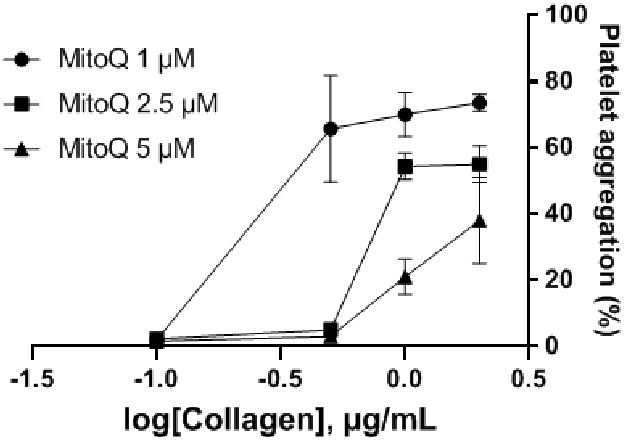

Figure 4. Platelet antiaggregant effect of MitoQ on increasing collagen concentrations (0.1, 0.5, 1 and 2 µg/mL).

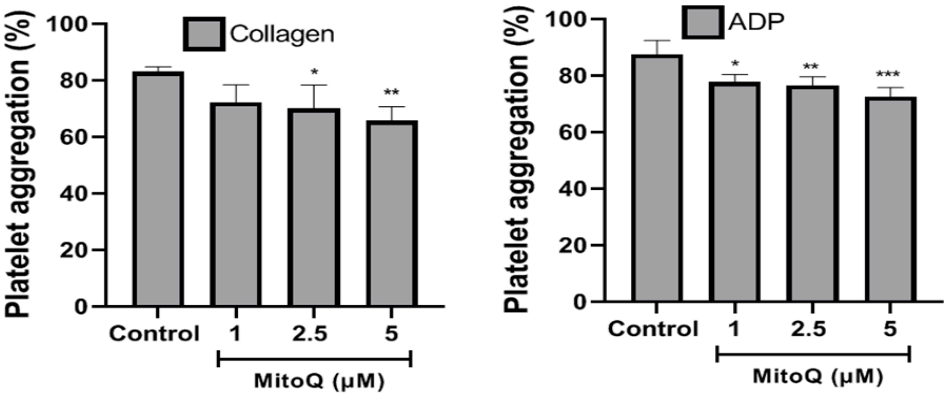

Figure 5. Platelet antiaggregant effect of MitoQ in Platelet-Rich Plasma stimulated by collagen 1 µg/mL or ADP 4 µM.

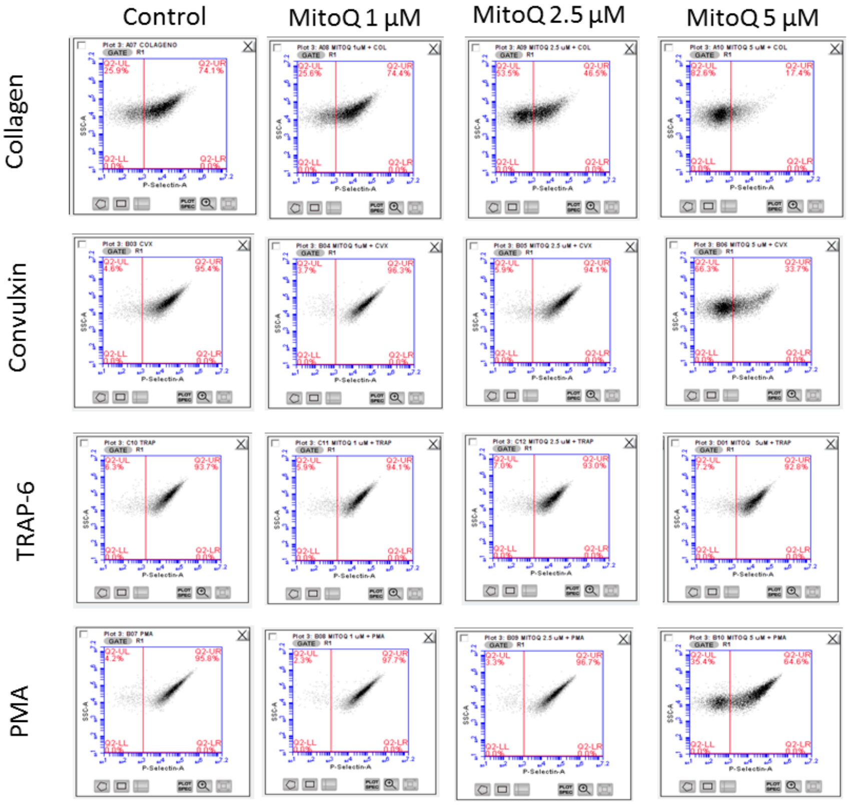

Figure 6. Representative dot plots of platelets P-selectin.

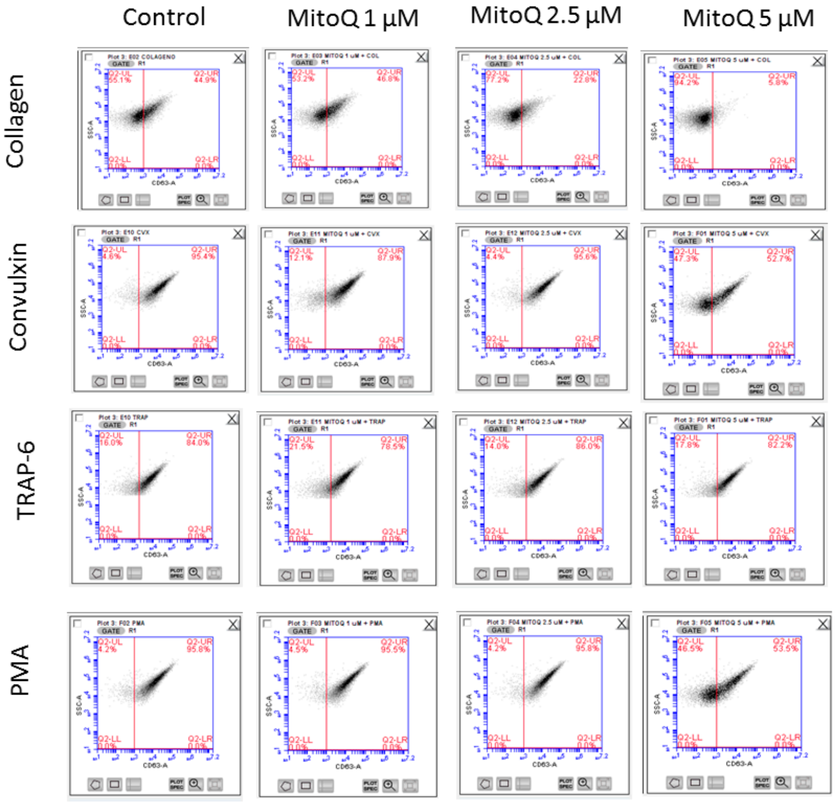

Figure S7. Representative dot plots of platelets CD63+.

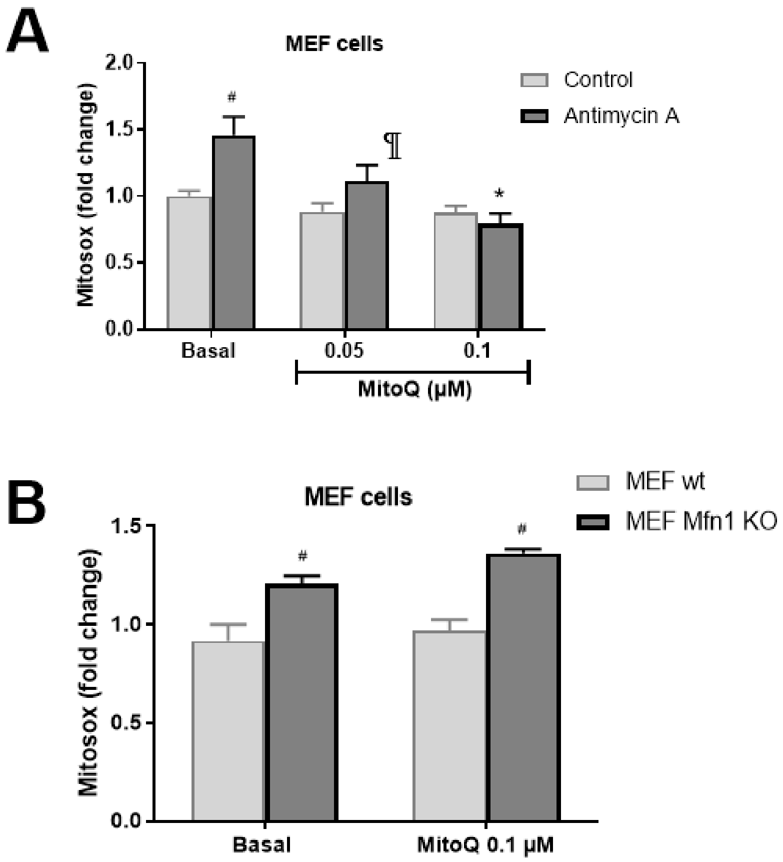

Figure 8. MitoQ decreases intraplatelet ROS in MEFwt and Mfn1KO cells. (A) and (B) Acute and chronic oxidative stress models in MEFwt and Mfn1KO cells. The statistical analysis was performed using the ANOVA (Tukey test). #$p<0.05$ vs basal (A) or MEF wt (B).

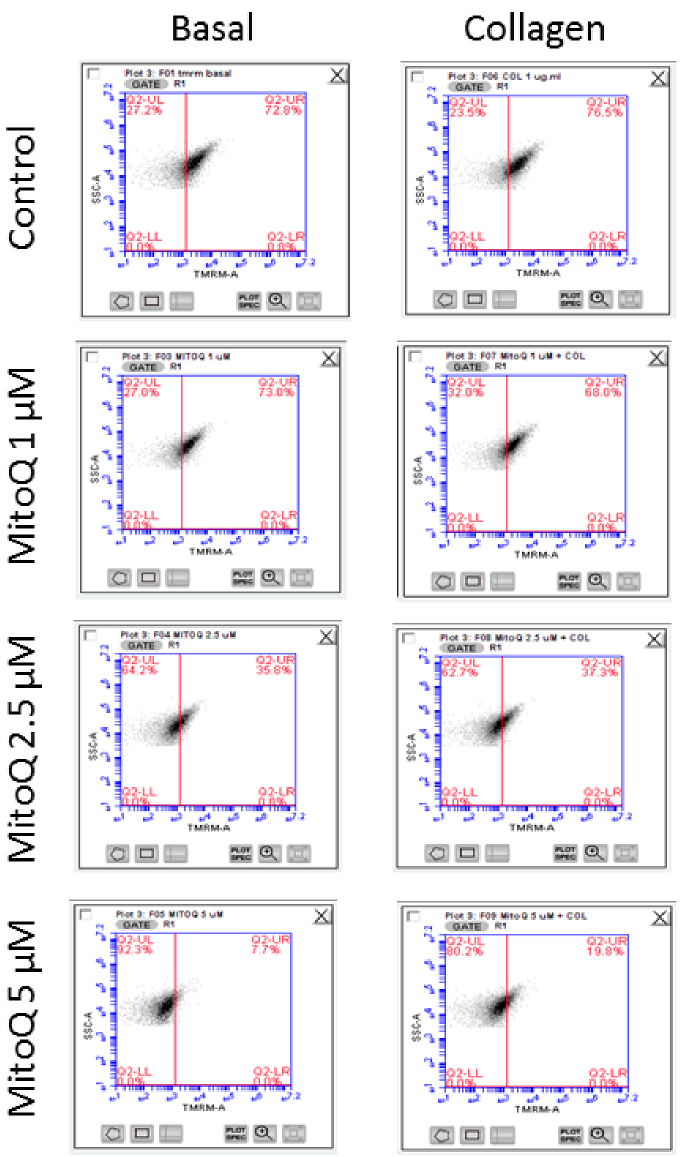

Figure 9. Effect of MitoQ on mitochondrial membrane potential (ΔΨm).

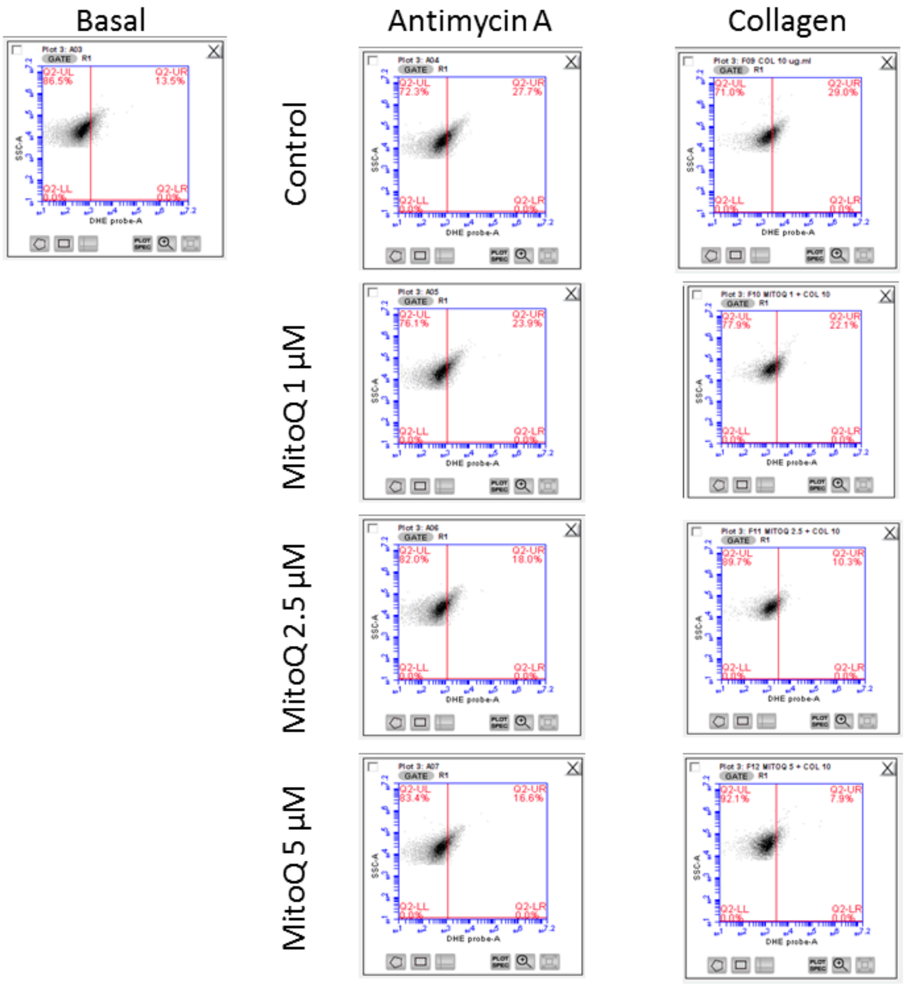

Figure S10. Effect of MitoQ on intraplatelet ROS generation. .
